# Navigating the Divide: A Comprehensive Review of the Mechanical and Anatomical Axis Approaches in Total Knee Replacement

**DOI:** 10.7759/cureus.57938

**Published:** 2024-04-09

**Authors:** Anmol Suneja, Sanjay V Deshpande, Gajanan Pisulkar, Shounak Taywade, Abhiram A Awasthi, Ankur Salwan, Sachin Goel

**Affiliations:** 1 Orthopedics, Jawaharlal Nehru Medical College, Datta Meghe Institute of Higher Education & Research, Wardha, IND; 2 Orthopedics and Traumatology, Jawaharlal Nehru Medical College, Datta Meghe Institute of Higher Education & Research, Wardha, IND

**Keywords:** personalized medicine, surgical outcomes, anatomical axis, mechanical axis, alignment approaches, total knee replacement

## Abstract

This comprehensive review explores the mechanical and anatomical axis approaches in total knee replacement (TKR) surgery, addressing the ongoing debate within the orthopedic community. Emphasizing the significance of TKR in alleviating knee-related disorders, this review underscores the pivotal role of accurate alignment in achieving optimal surgical outcomes. The purpose is to navigate the divide between the well-established mechanical axis approach, focusing on a straight-line alignment, and the anatomical axis approach, aligning with natural knee landmarks. The analysis delves into the advantages, disadvantages, and clinical implications of each approach, offering a nuanced perspective on their efficacy. The conclusion emphasizes a patient-centric approach, recommending the adoption of hybrid strategies and the incorporation of emerging technologies for enhanced precision. The future of TKR aligns with personalized medicine, leveraging advancements in computer-assisted navigation, robotics, and patient-specific implants. Ongoing professional development and interdisciplinary collaboration are crucial for surgeons, and as the field evolves, innovations in artificial intelligence, imaging, and 3D printing are expected to shape the trajectory of TKR alignment approaches.

## Introduction and background

Total knee replacement (TKR) stands as a transformative surgical intervention for individuals grappling with debilitating knee conditions, such as osteoarthritis or rheumatoid arthritis. The growing prevalence of these disorders, coupled with an aging population, has contributed to an increasing demand for TKR procedures globally [1]. The significance of TKR lies not only in its ability to alleviate pain and enhance joint function but also in its potential to restore the quality of life for those affected. Patients undergoing TKR often experience a substantial improvement in mobility, reduced pain, and an enhanced ability to engage in daily activities, making it a pivotal intervention in orthopedic medicine [2].

Accurate alignment during TKR is crucial for optimizing outcomes and ensuring the long-term success of the procedure. The alignment of the prosthetic components directly influences joint biomechanics, stability, and overall implant longevity. As such, achieving precise alignment is a cornerstone in the pursuit of successful TKR outcomes [3]. In the landscape of TKR, two predominant approaches have emerged in the quest for optimal alignment: the mechanical axis approach and the anatomical axis approach. This review seeks to delve into the intricacies of these divergent methodologies, aiming to shed light on their respective merits, drawbacks, and clinical implications [4]. The field of orthopedic surgery has witnessed a longstanding debate regarding the ideal axis alignment strategy in TKR. The divide between the mechanical and anatomical axis approaches has sparked discussions among surgeons worldwide. This review endeavors to navigate this divide, critically examining the rationale behind each approach and fostering a deeper understanding of their implications [3]. To provide a thorough assessment, this review undertakes a comprehensive evaluation of both mechanical and anatomical axis approaches. By scrutinizing the surgical techniques associated with each method, exploring the advantages and disadvantages, and delving into the outcomes reported in clinical studies, a nuanced perspective on the efficacy of these alignment strategies in TKR will be presented. Through this exploration, we aim to contribute valuable insights to the ongoing discourse within the orthopedic community.

## Review

Mechanical axis approach

Definition and Explanation

Mechanical axis concept in TKR: In TKR, the mechanical axis is defined by a line drawn from the center of the femoral head to the center of the ankle joint. Maintaining the limb's axis at 180º ± 3º is crucial in TKR, as studies have linked this alignment with enhanced clinical outcomes. The mechanical axis approach is one of the three alignment strategies in TKR, alongside the anatomical and kinematic axes. While the mechanical axis focuses on preserving the limb's axis within the specified range, the anatomical axis aims to replicate the native knee anatomy, and the kinematic axis aims to restore the native joint line, often considering the common occurrence of constitutional varus. The mechanical axis approach enjoys widespread adoption in TKR due to its consistent reproducibility, although ongoing research explores its long-term implications and safe alignment ranges [3-5].

Surgical techniques for mechanical axis alignment: Surgical techniques for achieving mechanical axis alignment in TKR involve using standard instrumentation and performing necessary soft-tissue releases to maintain the limb's axis within the specified range of 180º ± 3º. Typically, an extramedullary guide executes a perpendicular cut (90°) relative to the tibia's long axis. Establishing femoral component rotation involves making a posterior femoral cut parallel to the femur's mechanical axis. Various methods, such as measured resection or gap balancing techniques, are employed to achieve desired femoral rotation targets [6-9]. Considerations include maintaining appropriate ligament tension, optimizing quadriceps function, and ensuring proper patella tracking. While newer technologies like navigation, patient-specific instrumentation (PSI), and robotic-assisted techniques offer alternative means to determine mechanical axis alignment, the conventional method using extramedullary guides remains prevalent. Ultimately, the objective of mechanical axis alignment is to attain neutral alignment, where the mechanical axis of the entire leg intersects with the center of the knee joint [8].

Advantages and Disadvantages

Precision of alignment: Achieving precise alignment in total knee arthroplasty (TKA) is pivotal for optimizing patient outcomes. Various approaches and technologies have been developed to enhance alignment precision in TKA, including personalized knee guides, KA, and robotic-assisted surgery. A study comparing the precision of bony resections during TKA, using different computer-assisted methods, found no statistically significant differences in age, sex, and BMI between groups. However, a minor yet statistically significant distinction in distal femoral resection precision was noted, although it was likely clinically insignificant [9]. Personalized knee guides have emerged as a cost-effective and accessible technology for achieving precise, personalized TKA alignment. There is growing recognition that achieving the desired alignment necessitates precise enabling technologies despite the mechanical axis approach traditionally being the standard for TKA. Techniques such as kinematic alignment (KA) and robotic-assisted surgery have been developed to enhance TKA alignment precision [6,8,10]. Continuous research efforts in this area focus on utilizing personalized guides, KA, and robotic-assisted surgery to refine TKA alignment precision, ultimately aiming to improve patient outcomes.

Potential complications and limitations: TKA is a surgical intervention to alleviate pain, restore function, and enhance the quality of life for patients with end-stage degenerative knee osteoarthritis. However, akin to any surgical procedure, TKA carries potential complications and limitations. Complications may encompass bleeding, wound complications, thromboembolism, neural deficits, vascular issues, medial collateral ligament injury, instability, and aseptic loosening, which stands as the primary cause of late TKA failure [11-13]. Furthermore, the utilization of robotic systems in TKA has been associated with complications such as pin-hole fractures, pin-related infections, iatrogenic soft-tissue and bony injuries, and excessive blood loss [14]. Limitations of TKA may include implant wear and tear, restricted range of motion, and the necessity for revision surgery in certain instances [8]. Patients must discuss with their healthcare provider the potential risks and benefits of TKA to make well-informed decisions.

Clinical Studies and Outcomes

Literature review: Numerous studies and reviews contribute to understanding alignment in TKA. One systematic review compared PSI KA to non-PSI mechanical alignment (MA) in TKA and found no significant differences in clinical outcomes between the two methods [15]. In a randomized controlled trial, functional alignment (FA) with bony resection balancing was compared to MA with soft-tissue release balancing using robotic arm-assisted technology, revealing similar patient outcomes for both approaches [16]. A review article surveyed alignment options in TKA, encompassing MA, KA, anatomical, and FA, emphasizing further investigation into safe limb alignment ranges and alignment's correlation with long-term functional outcomes and survivorship [17]. Additionally, Orthobullets provided insights into the mechanical axis of the femur, defined as a line linking the center of the femoral head to the point where the anatomic axis meets the intercondylar notch [18]. These findings underscore the importance of alignment in TKA yet highlight the need for tailored approaches and ongoing research to optimize long-term clinical results.

Comparative analysis of mechanical axis outcomes: Various studies have scrutinized the outcomes of mechanical axis alignment in TKA. One investigation aimed to evaluate the reliability of conventional instrumentation in achieving intended femoral and tibial coronal alignment [19]. Another study compared KA with MA to assess their impact on knee function and clinical outcomes [20]. Additionally, a prospective randomized control trial protocol was devised to compare functional with mechanical axis alignment in TKA, yielding diverse results favoring either KA or MA [21]. Furthermore, a study emphasized that solely targeting MA may lead to unfavorable kinematic consequences without appropriately positioned femoral and tibial components [3]. Lastly, an early clinical comparative study assessed TKA outcomes with KA using specific instruments versus MA in varus knees [22]. These studies provide insights into the efficacy and implications of mechanical axis alignment in TKA, informing clinical decision-making and future research directions.

Anatomical axis approach

Definition and Explanation

Anatomical landmarks in TKR: TKR relies on several crucial anatomical landmarks, including the posterior cruciate ligament (PCL), the "Akagi line," and various bony structures such as the medial and lateral epicondyles, tibial tubercle, fibular head, and inferior pole of the patella. These landmarks are pivotal in determining the rotational alignment of the tibial component and calculating the ideal joint line position. Among them, bony landmarks stand out for their reliability. They are commonly utilized in clinical settings for TKR procedures due to their consistent references, ensuring precise alignment and restoration of the joint line. Particularly in revision TKR or cases involving severe bone loss, these anatomical landmarks are indispensable to guarantee optimal implant positioning and overall procedural success [23].

Surgical techniques for anatomical axis alignment: In TKA, achieving anatomical axis alignment involves specific surgical techniques, including utilizing extramedullary guides for the tibia to execute a perpendicular cut and positioning components to mimic the true anatomy of the femur and tibia closely. Advanced methods for determining the mechanical axis, such as navigation, PSI, and robotic-assisted techniques, also contribute to achieving anatomical axis alignment. Furthermore, setting the rotation of the femoral component to the posterior condylar axis is instrumental in attaining anatomic alignment [7]. The anatomical axis approach focuses on restoring the natural alignment of the knee and has demonstrated improved clinical outcomes in select studies [4,6].

Advantages and Disadvantages

Natural joint biomechanics: The intricate dynamics of natural joint biomechanics encompass the coordinated interaction among articular surfaces, ligaments, and muscles, ensuring mobility, stability, and adequate load transmission. Research endeavors, such as those exploring the biomechanics of the human ankle joint, shed light on vital aspects, including passive structures, the axis of joint rotation, and the pivotal role of ligaments in guiding and stabilizing joint movement [24]. Biomechanics is a cornerstone for comprehending the forces acting on bones, joints, and muscles during everyday activities, profoundly influencing orthopedic health and developing prosthetics, surgical methodologies, and treatment strategies [25].

Challenges and limitations: The anatomical axis approach in TKA presents notable challenges and drawbacks, primarily stemming from the complexity of accurately identifying reproducible anatomical landmarks, which can potentially lead to alignment errors. Moreover, this approach may not be universally applicable, particularly for patients with severe deformities or ligamentous instability. Implementing the anatomical axis approach may necessitate specialized techniques and instrumentation, augmenting the surgical procedure's intricacy [15,26,27]. Surgeons must diligently assess each patient's unique condition and carefully consider the potential constraints of the anatomical axis approach when determining the most suitable alignment technique for TKA.

Clinical Studies and Outcomes

Examination of relevant research: Several investigations have delved into the anatomical axis approach in TKA, offering valuable insights into its efficacy and implications. One study aimed to establish a pure, unbiased, reliable, and precise objective relationship among local knee axis measurements [28]. Another comparative study scrutinized the clinical outcomes of KA versus MA in TKA, revealing that proponents of KA emphasize bone preservation, reduced postoperative pain, and enhanced postoperative function, thereby diminishing the proportion of dissatisfied patients post-TKA [29]. A systematic review examining alignment options for TKA underscored that anatomical alignment strives to replicate the native knee anatomy and geometry while considering the inherent varus angulation of the proximal tibial plateau [15]. Additionally, another review advocated for future studies on TKA alignment to leverage surgical adjuncts (e.g., robotic technology) to enhance alignment accuracy, incorporate intraoperative evaluations of knee biomechanics and periarticular soft-tissue tension, and correlate alignment with long-term functional outcomes and survivorship [17], despite some studies suggesting potential benefits of anatomical alignment, such as improved pain relief and functional outcomes, consensus regarding the optimal alignment for arthroplasty function and results still need to be discovered. Thus, further research is warranted to ascertain the long-term clinical outcomes and implant survival associated with the anatomical axis approach.

Comparison of anatomical axis outcomes across studies: The comparison of anatomical axis outcomes unveils notable variations in the relationship between the anatomical and mechanical axes, particularly in diverse lower extremity deformities. For instance, a study uncovered a correlation coefficient of 0.685 between the anatomical and mechanical axes for varus knees, diverging from previous findings and indicating a distinct relationship [28]. Furthermore, another study emphasized the importance of considering anatomical and mechanical axes within the broader context of lower extremity alignment, particularly concerning procedures like TKAs and femoral fracture fixations [30]. These findings underscore the intricate nature of anatomical axis outcomes, highlighting the nuanced interplay between various factors and the imperative for further research to elucidate their implications for orthopedic procedures. As such, a comprehensive understanding of anatomical axis alignment's complexities is crucial for optimizing surgical outcomes and patient care in orthopedic practice.

Navigating the divide: Comparative analysis

Overview of the Divide Between the Mechanical and Anatomical Axis Approaches

The divergence between the mechanical and anatomical axis approaches in TKR stems from the distinct alignment techniques employed for implant positioning. MA strives to maintain the limb axis within 180º ± 3º, whereas anatomical alignment aims for neutral alignment with a slight 2-3 varus joint line relative to the mechanical axis [6]. In contrast, KA seeks to replicate the native knee anatomy and geometry, accommodating the inherent varus angulation of the proximal tibial plateau [31]. Consensus regarding the superior alignment approach for arthroplasty function and outcomes remains elusive [32]. While MA promotes knee flexion and uniform component wear, it imposes an unnatural limb position that alters knee biomechanics [31]. Conversely, KA may improve pain relief and functional outcomes [32]. Nonetheless, no significant disparities are observed in the postoperative complications, changes in hemoglobin levels, length of hospital stay, hip-knee-ankle angle, joint line orientation, or the overall functional outcomes between the KA and MA techniques [31]. The selection of an alignment approach should carefully consider the patient's specific condition and expectations as well as the available surgical techniques and technologies [3]. Using robotic and computerized navigation systems can enhance the precision of planned alignment execution [31].

Factors Influencing the Surgeon's Choice

**Figure 1:**
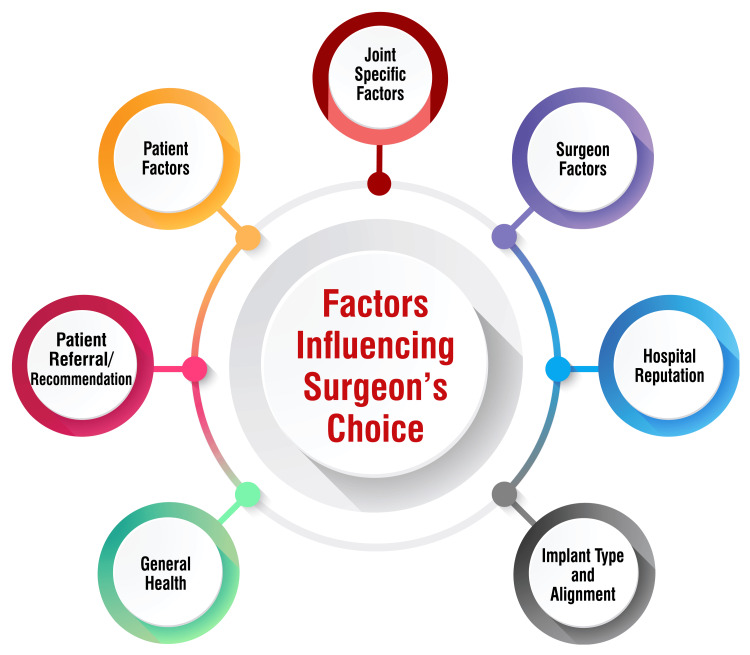
Factors influencing the surgeon's choice Image Credit: Dr. Anmol Suneja

Patient factors such as age, sex, expectations, socioeconomic status, and comorbidities significantly influence the selection of an alignment approach [33]. Additionally, joint-specific factors, including whether the replacement is for the hip or knee, whether it's total or partial, the pattern of disease (varus or valgus), and specific alignment preferences of the joint are crucial considerations [34]. Surgeon-related factors such as the volume of TKR procedures performed, surgical technique, and experience level also weigh heavily in the decision-making process [34]. Moreover, hospital reputation can impact a surgeon's choice of alignment approach, as surgeons may opt for approaches based on the hospital's reputation [35]. Patient referral or recommendation by a medical doctor appears to be a pivotal factor influencing a joint replacement surgeon's choice of alignment approach [36]. Implant type and alignment preferences also come into play, as surgeons may select implants based on cost, quality, and alignment preferences [37]. Additionally, general health considerations, including comorbidities, frailty, and the overall quality of life, are essential in determining the most suitable alignment approach [38]. It is imperative to consider these multifaceted factors when selecting the appropriate alignment approach for a patient, as it can profoundly impact the patient's satisfaction, functional outcomes, and overall quality of life following TKR. Factors influencing the surgeon's choice are shown in Figure 1.

Patient-Specific Considerations

In knee replacement surgery, patient-specific considerations encompass the individual's unique condition and expectations and the array of surgical techniques and technologies. Introducing patient-specific approaches to TKA, preoperative imaging techniques such as plain radiographs, computed tomography, and magnetic resonance imaging are employed to craft cutting blocks tailored to the patient's anatomy [39]. These patient-matched cutting blocks offer potential advantages, including reduced operative time, minimal instrument trays required, and the ability to meticulously plan components' size, position, and alignment before surgery [39]. Nevertheless, several factors must be considered when implementing patient-specific knee arthroplasty. This includes ensuring meaningful patient-specific outcomes, assessing whether the implant can lead to a more stable knee during flexion, evaluating the potential to streamline operative and setup times, and optimizing the utilization of operating rooms and hospital spaces [40]. Ultimately, the alignment approach in knee replacement surgery should thoroughly account for the patient's specific condition and expectations while considering the available surgical techniques and technologies [31].

Hybrid Approaches

Hybrid approaches in TKR merge elements from MA and anatomical alignment techniques, striving to harness the advantages of each while mitigating their limitations. An example of such a hybrid method involves integrating computer navigation with traditional TKR procedures. A study in the Journal of Orthopaedic Surgery and Research elucidated a hybrid navigation technique that blends the advantages of computer navigation with conventional TKR methodologies [41]. This approach leverages navigation systems to provide insights on hardware presence, extra-articular deformities, and bone loss while aiming to achieve a final mechanical axis of 0°, allowing for up to 3° of varus or valgus malalignment overall [41]. Furthermore, a comparative study scrutinized KA and MA techniques in primary TKR, revealing that the KA approach resulted in notably shorter operation times and superior overall functional outcomes compared to the MA technique. Interestingly, the KA technique yielded better results despite placing the femoral component slightly more valgus than the mechanical axis and the tibial component slightly more varus [31]. In essence, hybrid approaches in TKR aspire to capitalize on the strengths of MA and anatomical alignment techniques while mitigating their shortcomings. Nevertheless, a consensus regarding the optimal alignment approach for arthroplasty function and outcomes still needs to be reached. Thus, when selecting an alignment technique, it is imperative to consider the patient's specific condition, expectations, and available surgical techniques and technologies. This holistic approach ensures that the chosen method aligns with the patient's needs and maximizes the potential for successful outcomes in TKR procedures.

Future directions and emerging technologies

Technological Advancements in TKR

Computer-assisted navigation is an emerging technology in TKR surgery that enhances the accuracy and precision of component alignment, soft-tissue protection, and postoperative outcomes. Introduced as an adjunct to TKA, computer-assisted navigation holds promise for improving the positioning and alignment of TKA components [42]. This navigation software facilitates accurate postoperative alignment by enabling precise and reproducible bony resection and ligament balancing [43]. Classical computer-assisted systems for TKR employ real-time surgical navigation using infrared optical tracking arrays [44]. By optimizing the precision and accuracy of the surgical procedure, computer-assisted navigation enhances the predictability of TKR outcomes, provided the correct target is identified [44]. Additionally, robotic-assisted TKA (RATKA) offers the added benefit of improving soft-tissue protection [45]. The available literature examines each system's accuracy and precision of component alignment, soft-tissue protection, postoperative outcomes, and related costs [45].

Robotics in TKR represents another innovative technology that enhances the accuracy and precision of component alignment, soft-tissue protection, and postoperative outcomes. RATKA has shown associations with improved early functional recovery and reduced time to hospital discharge compared to conventional TKA [46]. While robotic-assisted TKA offers enhanced functionality compared to computer navigation, the substantive advantages over navigation systems are yet to be firmly established [47]. Computer-assisted navigation and robotic technology can optimize the surgical procedure's precision and accuracy, leading to more predictable outcomes in TKR when the correct target is identified [42,45]. The literature examines the accuracy and precision of component alignment, soft-tissue protection, postoperative outcomes, and associated costs for each system [45].

Personalized Medicine in TKR

Patient-specific implants represent a significant advancement in personalized medicine within TKR, aiming to enhance the accuracy of implant placement and alignment. These systems utilize preoperative imaging to create customized surgical guides and implants tailored to each patient's anatomy to restore the native knee anatomy and physiological soft-tissue laxity [48,49]. By replicating the unique knee geometry of individuals, which can vary by factors such as gender, ethnicity, and body type, personalized implants offer potential advantages. However, their widespread implementation may need to be improved by factors like cost and production time [50]. Nonetheless, personalized medicine is gaining traction in orthopedics, including TKR, where, despite various initiatives, many patients still need to be satisfied [49]. Thus, while offering promising benefits, personalized medicine in TKR should carefully consider the patient's specific condition and expectations and the available surgical techniques and technologies.

Precision medicine in alignment strategies for TKR seeks to restore the native knee anatomy and accommodate the natural variability of knee phenotypes [48,49]. Utilizing personalized surgical guides, implants, and adjuncts like robotic technology can enhance the precision of implant placement and alignment [51]. The paradigm shift from MA toward a greater understanding of knee phenotype variability has propelled the adoption of personalized TKR [49]. However, further scientific evidence is needed to fully support the implementation of personalized medicine in orthopedics [51]. Consequently, the choice of alignment strategy in TKR should consider the patient's specific condition and expectations alongside the available surgical techniques and technologies, ensuring optimal outcomes for each individual.

## Conclusions

In conclusion, examining the mechanical and anatomical axis approaches in TKR reveals a complex landscape with distinct advantages and challenges for each strategy. The mechanical axis approach, emphasizing a straight-line alignment, provides consistency and simplicity in execution yet raises concerns about potential complications. Conversely, the anatomical axis approach, aligning with natural landmarks, offers potential biomechanical advantages but introduces challenges related to patient variability. Surgeons are encouraged to adopt a patient-centric approach, considering individual anatomical variations and clinical characteristics. Hybrid approaches, combining elements of both strategies and embracing emerging technologies like computer-assisted navigation and robotics, are recommended for enhancing precision. Ongoing professional development, interdisciplinary collaboration, and staying informed about evolving trends in TKR are crucial for surgeons. Looking ahead, the future of TKR aligns with personalized medicine, with advancements in technology, patient-specific implants, and innovative approaches aimed at tailoring interventions to individual anatomy and biomechanics. Integrating artificial intelligence, advanced imaging techniques, and 3D printing holds promise for refining our understanding of knee biomechanics and shaping the future of TKR alignment approaches.
